# Regulation of Cardiac Conduction and Arrhythmias by Ankyrin/Spectrin-Based Macromolecular Complexes

**DOI:** 10.3390/jcdd8050048

**Published:** 2021-04-29

**Authors:** Drew Nassal, Jane Yu, Dennison Min, Cemantha Lane, Rebecca Shaheen, Daniel Gratz, Thomas J. Hund

**Affiliations:** 1 The Frick Center for Heart Failure and Arrhythmia, The Dorothy M. Davis Heart and Lung Research Institute, The Ohio State University, Columbus, OH 43210, USA; drew.nassal@osumc.edu (D.N.); yu.2164@buckeyemail.osu.edu (J.Y.); min.196@buckeyemail.osu.edu (D.M.); lane.532@osu.edu (C.L.); shaheen.79@osu.edu (R.S.); gratz.24@osu.edu (D.G.); 2 Department of Biomedical Engineering, College of Engineering, The Ohio State University, Columbus, OH 43210, USA; 3 Department of Internal Medicine, College of Medicine, The Ohio State University Wexner Medical Center, Columbus, OH 43210, USA

**Keywords:** pacemaking, arrhythmia, ankyrin, spectrin

## Abstract

The cardiac conduction system is an extended network of excitable tissue tasked with generation and propagation of electrical impulses to signal coordinated contraction of the heart. The fidelity of this system depends on the proper spatio-temporal regulation of ion channels in myocytes throughout the conduction system. Importantly, inherited or acquired defects in a wide class of ion channels has been linked to dysfunction at various stages of the conduction system resulting in life-threatening cardiac arrhythmia. There is growing appreciation of the role that adapter and cytoskeletal proteins play in organizing ion channel macromolecular complexes critical for proper function of the cardiac conduction system. In particular, members of the ankyrin and spectrin families have emerged as important nodes for normal expression and regulation of ion channels in myocytes throughout the conduction system. Human variants impacting ankyrin/spectrin function give rise to a broad constellation of cardiac arrhythmias. Furthermore, chronic neurohumoral and biomechanical stress promotes ankyrin/spectrin loss of function that likely contributes to conduction disturbances in the setting of acquired cardiac disease. Collectively, this review seeks to bring attention to the significance of these cytoskeletal players and emphasize the potential therapeutic role they represent in a myriad of cardiac disease states.

## 1. Introduction

Over an average lifetime, the heart beats more than 2 billion times with very little margin for failure. Adding to this challenge, heart rate must adapt to acute and chronic changes in physiologic demands to maintain effective cardiac output. Thus, generation of the heartbeat requires a highly coordinated system that is both durable and flexible. An elaborate cardiac conduction system, comprised of excitable tissues responsible for initiation and organized spread of the electrical impulse, provides the necessary support for the daunting task of reliable control of heart rate and rhythm. Dysfunction of the cardiac conduction system is a common cause of cardiac arrhythmia leading to dyspnea, syncope, fatigue, heart failure symptoms, chest pain, or cardiac arrest, often resulting in cardiac pacemaker implantations at the rate of more than 1 million each year around the world [1].

The cardiac conduction system may be roughly divided into an electrical impulse generation component (primarily the sinoatrial node) and impulse conducting component (e.g., atrioventricular node and His-Purkinje system). Electrophysiological properties are highly heterogeneous throughout the conduction system reflecting variable ion channel expression and tissue structure [2]. Not surprisingly, a large number of acquired or inherited defects in ion channels expressed in myocytes in the conduction system have been associated with cardiac conduction/rhythm disturbances and arrhythmia [3,4,5]. While the majority of therapeutic approaches for addressing heart rhythm or conduction defects attempt to target specific channels and their function, there is growing appreciation for the importance of associated cytoskeletal and adapter proteins in maintaining normal ion channel function [6,7,8,9]. We aim with this review to highlight the role of the underlying cytoskeletal elements, particularly ankyrin and spectrin polypeptides, in organizing macromolecular complexes for regulation of ion channel activity and membrane excitability in myocytes throughout the cardiac conduction system.

## 2. The Cardiac Conduction System in Health and Disease

**Figure 1:**
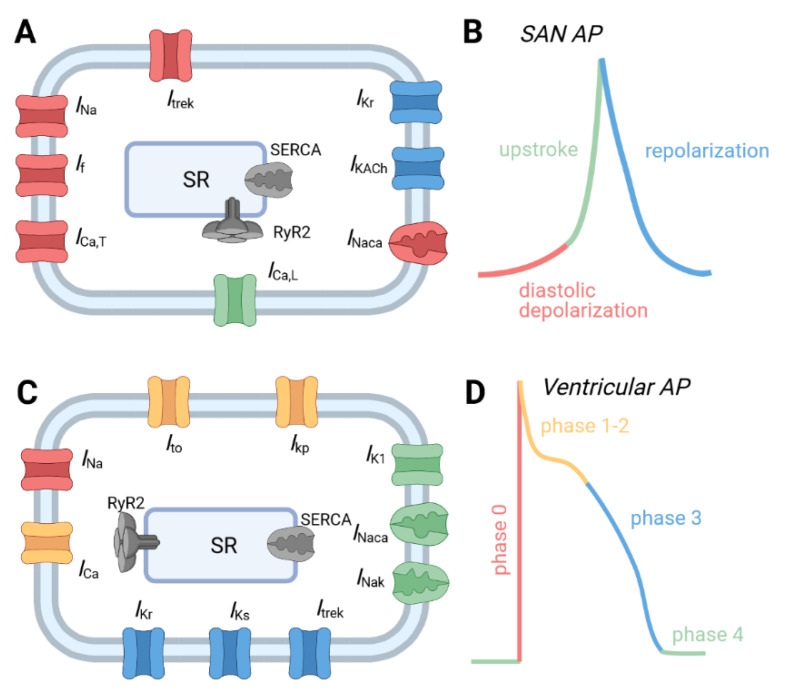
Major ion channels underlying the action potential from different regions of the cardiac conduction system. (**A**) Schematic of major ion channels and representative action potentials from (**A**,**B**) sinoatrial node and (**C**,**D**) ventricular myocytes. Channels are color coded to correspond to phase of the AP where they make an important contribution. Abbreviations are as follows: Acetylcholine-activated K^+^ current (*I*_KAch_); Inward rectifier K^+^ current (*I*_K1_); L-type Ca^2+^ current (*I*_Ca_); Na^+^/Ca^2+^ exchanger (*I*_NaCa_); Na^+^/K^+^ ATPase (*I*_NaK_); plateau K^+^ current (*I*_Kp_); rapid (*I*_Kr_) and slow (*I*_Ks_) delayed rectifier K^+^ currents; sarcoplasmic reticulum (SR); SR Ca^2+^ release channel (RyR2); SR Ca^2+^ ATPase (SERCA); transient outward K^+^ current (*I*_to_); TWIK-related K^+^ channel 1 current (*I*_trek_); T-type Ca^2+^ current (*I*_Ca,T_); voltage-gated Na^+^ current (*I*_Na_).

Proper function of the cardiac conduction system depends on coordinated activity of a heterogeneous collection of excitable cells distributed throughout the heart. In turn, normal myocyte excitability depends on orchestrated function of a diverse assortment of ion channels (Figure 1). The myocyte maintains tight spatio-temporal control over ion channel function through organization of ion channel macromolecular complexes involving a host of adapter, cytoskeletal and regulatory molecules [6,10]. While defects in ion channels themselves are closely linked to cardiac conduction disturbances and arrhythmia, there is growing evidence that disruption of associated adapter/cytoskeletal proteins also underlies cardiac conduction system dysfunction.

### 2.1. Normal Physiology of the Cardiac Conduction System

The normal heartbeat begins with a spontaneous electrical impulse generated by the sinoatrial node (SAN), a specialized crescent-shaped tissue less than an inch in length located in the wall of the right atrium adjacent to the opening of the superior vena cava. From the SAN, activation spreads through the atria and to the atrioventricular node (AVN), which serves as a gateway for electrical activation between the atria and ventricles. The impulse then travels from the AVN to the ventricles through the His-Purkinje system comprised of the AV bundle, right and left bundle branches that run along the interventricular septum and finally Purkinje fibers that allow the action potential to spread rapidly in an organized fashion through the ventricular free walls. Each component of the cardiac conduction system is imbued with unique electrophysiological and structural features to support its specific role. For example, the SAN, unlike the majority of cardiac tissue, does not have a stable resting membrane potential, but instead generates spontaneous firing through cyclical depolarizing events. Changes in the firing rate of the SAN node contribute to changes in heart rate to meet changing physiologic demands of the body. The AVN also displays automaticity but at an intrinsic rate slower than the SAN allowing it to serve in a backup capacity for pacemaking in the event of SAN failure. The AVN supports very slow conduction, which helps insulate the ventricles in the case of rapid, arrhythmic activity in the atria. Purkinje myocytes support extremely rapid conduction and are bundled in insulated fibers to prevent premature or inappropriate ventricular activation. Purkinje-muscle junctions allow for coupling with slow and safe conduction to prevent propagation failure resulting from source-sink mismatch between the small, current generating volume of the Purkinje fiber and very large surrounding ventricular mass.

### 2.2. Cardiac Conduction System Dysfunction and Disease

Generation of the cardiac action potential within these various subregions of the heart and the electrical propagation between them is the result of the coordination of an array of Na^+^, K^+^ and Ca^2+^ ion channels. Mutations resulting in both gain- and loss-of-function changes in these ion transport proteins are not only sufficient to generate arrhythmic dysfunction in both rhythm and rate of the heart, but have been critical to understanding the function of for each of these players. Importantly, inherited and acquired defects in a host of ion channels have been identified in myocytes throughout the conduction system and are linked to various descriptions of cardiac arrhythmia, resulting in defects in cardiac automaticity and/or conduction [5]. Specific disruptions to heart rate include SAN dysfunction (also referred to as sinus node disease [SND] or sick sinus syndrome [SSS]) where the primary pacemaker is unable to generate appropriate heart rates for the physiologic demands of the body. This includes sinus bradycardia and tachycardia, sinus arrest, sinus-exit block, or chronotropic incompetence, leading to instances of exercise intolerance or syncope [11,12]. Development of such conditions can be the outcome of both genetic causes as well as acquired states, often being induced from electrical and structural remodeling associated with pathologic states such as atrial fibrillation, heart failure, ischemia, or simply aging. Acquired dysfunction may also be the result of drug intoxication from a variety of compounds, including β-blockers, Ca^2+^ channel blockers and digitalis [3]. Other rate disorders can arise from AV node block, which occurs when there is partial or complete block of impulse propagation through the AVN, resulting in delays between atrial and ventricular tissue in mild cases, or complete uncoupling in the most severe. Alternatively, a condition known as Wolff-Parkinson-White (WPW) syndrome, describes a state of early excitation of the ventricles through an accessory pathway that bypasses the slow conduction of the AV node, shortening the interval between atrial and ventricular excitation, resulting in tachycardia and impairment of proper filling of the ventricular chambers.

In addition to rate disturbances, cardiac conduction system dysfunction can result in abnormal rhythm arising from changes in myocyte excitability and/or intercellular communication that impact the ability of the electrical impulse to propagate through the heart. Disruption to conduction can lead to conduction disease, which includes left and right bundle branch block, causing asynchronous contraction of the ventricles. More severe consequences of conduction disorders are arrhythmias, including atrial flutter/fibrillation and ventricular tachycardia/fibrillation. Such arrhythmias can arise from disruption to normal states of conduction but are also the consequence of genetic or acquired remodeling of the cardiac action potential, including long QT syndrome, short QT syndrome, Brugada syndrome, arrhythmogenic cardiomyopathy, and catecholaminergic polymorphic ventricular tachycardia (CPVT). These conditions can provide pathologic substrates for creating arrhythmias by impacting the dynamics of cellular excitability or providing their own excitation decoupled from normal sinus rhythm. Despite appreciation for the association between disruption of ion channel function/expression and conduction disease, the mechanistic underpinnings, especially in acquired disease, are not fully understood. At the same time, there is growing appreciation that aside from primary defects in ion channels, dysfunction of associated adaptor and cytoskeletal proteins give rise to arrhythmia. In particular, mounting data support a critical role for ankyrin and spectrin proteins in organizing ion channel macromolecular complexes in myocytes throughout the cardiac conduction system with strong link to inherited and acquired forms of disease. Moreover, these adapter complexes have been identified to regulate a range of ion channel targets, suggesting their potential influence to induce and/or restore proper balance of cardiac electrophysiology targets. 

## 3. Ankyrins in the Cardiac Conduction System

Ankyrins are adapter proteins that link membrane proteins (e.g., ion channels and transporters, cell adhesion molecules, and signaling proteins) to the actin/spectrin-based cytoskeleton, ultimately serving to establish stable membrane expression for interacting proteins. The family is comprised of three members encoded by distinct genes: ankyrin-R (encoded by *ANK1*), ankyrin-B (encoded by *ANK2*), and ankyrin-G (encoded be *ANK3*). Ankyrin isoforms are expressed throughout the body, including kidney, lung, brain, skeletal muscle, and heart with ankyrin-R showing the most restricted expression, being found primarily in erythrocytes with secondary expression in muscle and neurons. Ankyrin-B and -G, on the other hand, are ubiquitously expressed, including in myocytes throughout the cardiac conduction system [13,14] and are the focus of our discussion here. 

**Figure 2:**
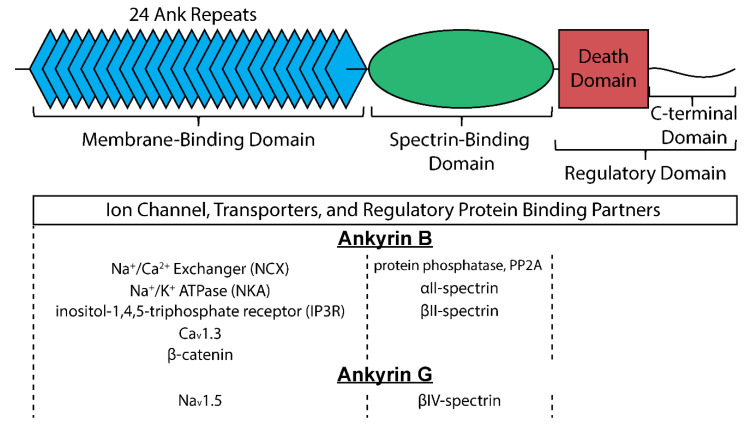
Structure of canonical ankyrin and major interacting proteins organized by site of interaction for ankyrin-B and ankyrin-G.

Canonical ankyrins are composed of four domains termed the membrane-binding domain (MBD), the spectrin-binding domain (SBD), the death domain (DD), and the C-terminal domain (CTD) (Figure 2) [15]. The MBD does not interact directly with the cell membrane but instead is primarily the site of interaction with membrane-bound proteins and is defined by a series of stacked α-helices coupled by β-hairpin loops (24 ANK repeats) [16], which generate specific binding pockets for membrane associated proteins. Importantly, and particularly relevant for its role as a multimodal regulator of cellular excitability, the MBD allows for simultaneous interaction with a number of membrane proteins facilitating formation of large protein complexes. Despite significant sequence homology of the MBD across ankyrin isoforms, isoform-specific protein-protein interactions are achieved at least in part through distinct subcellular localization profiles. In cardiac myocytes for example, ankyrin-B localizes to T-tubules, the sarcoplasmic reticulum (SR), and lateral plasma membrane [17], while ankyrin-G is found primarily at the intercalated disc membrane where neighboring cells are electrically and mechanically coupled [18]. As its name implies, the SBD is the site for interaction with β-spectrin isoforms, linking ankyrin with the actin-based cytoskeleton to stabilize large protein complexes [19]. The last two domains (DD and CTD) are collectively referred to as the regulatory domain for their role in modulating ankyrin interactions. Residues within this domain are important for intermolecular interactions and localization of associated membrane proteins [20,21,22]. Moreover, the majority of disease-causing mutations in ankyrin genes are located within the regulatory domain, supporting the central role of ankyrins as hubs for macromolecular complexes [23]. While the many associated mutations in ankyrin have allowed identification of its interacting partners and functional impacts [14,23], it is critical to realize that acquired states of heart disease associate with reductions in ankyrin expression, suggesting that disruption of each of these interacting partners can be impacted by loss in ankyrin expression [24,25,26,27]. While this provides a strong rationale for understanding the pathologic remodeling of electrical activity in the diseased heart, it also illustrates the profound therapeutic potential that may exist in achieving ways of preserving ankyrin expression. Currently, it is unclear what mechanisms account for the observed loss in ankyrin expression with disease, whether it is a transcriptionally mediated response, or pathways affecting protein stability and/or localization. Undoubtedly, however, unraveling such mechanisms with the intent to preserve ankyrin expression in heart disease will represent a therapeutic approach with significant and wide reaching implications for many of the observed states of electrical disruptions. The following discussion of what these interacting partners are and their consequence on heart rate and rhythm will hopefully illustrate the profound role these cytoskeletal proteins serve in maintaining proper cardiac electrical activity.

### 3.1. Ankyrin-B

**Figure 3:**
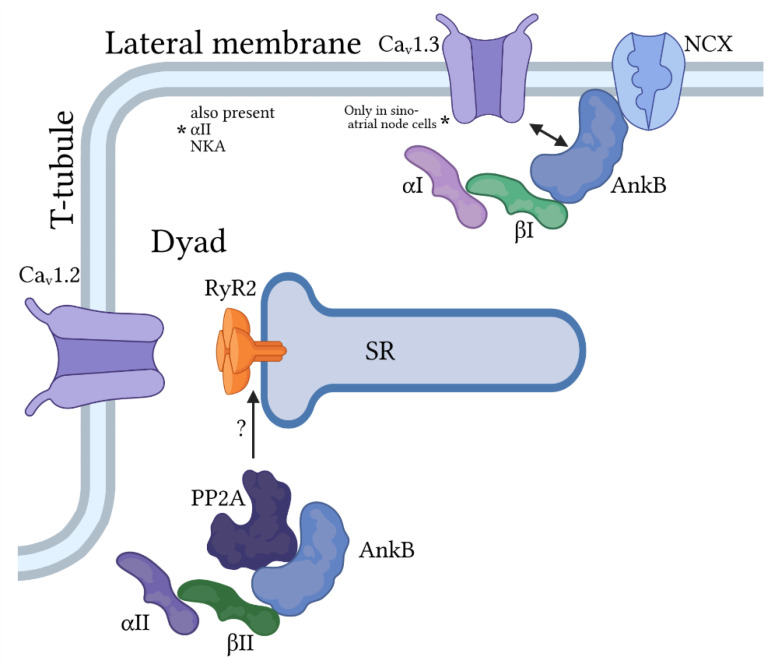
Ankyrin-B/spectrin-based macromolecular complexes for regulation of pacemaking and membrane excitability in cardiac myocytes. Ankyrin-B (AnkB) associates primarily with β_I_ and α_I_ or α_II_-spectrin at the lateral membrane to target the Na^+^/Ca^2+^ exchanger (NCX) and Na^+^/K^+^ ATPase and, in sinoatrial node cells only, the voltage-gated Ca^2+^ channel Ca_v_1.3. AnkB also associates with α_II_- and β_II_-spectrin to regulate proteins at the cardiac dyad formed at the juxtaposition of the t-tubule (location of voltage-dependent Ca^2+^ channel Ca_v_1.2) and sarcoplasmic reticulum (SR) membranes. Importantly, this AnkB/spectrin-based complex is important for regulation of Ca^2+^ cycling by the protein phosphatase PP2A, in part through modulation of the SR Ca^2+^ release channel RyR2.

Ankyrin-B is expressed within the nodal cells, the cardiac conduction system, and atrial and ventricular myocytes, where it interacts with several channels and transporters important for Ca^2+^ handling in cardiomyocytes, including the Na^+^/Ca^2+^ exchanger (NCX), Na^+^/K^+^ ATPase (NKA) and inositol-1,4,5-triphosphate receptor (IP_3_R) [15,28] as well as the protein phosphatase, PP2A (Figure 2 and Figure 3) [29,30]. In SAN myocytes, ankyrin-B has also been found to associate with Ca_v_1.3, a predominantly neuronal L-type Ca^2+^ channel that is an important determinant of spontaneous diastolic depolarization phase of the SAN action potential [31]. A human variant in ankyrin-B was first identified in a proband with prolongation of the QT interval on the electrocardiogram together with ventricular arrhythmias and sudden death, leading to the initial link to long QT syndrome (LQT type 4) [32]. It is now appreciated that loss of ankyrin-B function produces a complex phenotype (termed ‘ankyrin-B syndrome’) including SAN, atrial, and ventricular defects [31,33,34]. Relevant to the cardiac conduction system, loss of ankyrin-B leads to aberrant SAN myocyte excitability and pacemaking, displayed by severe bradycardia and rate variability, secondary to defects in Ca^2+^ cycling and loss of Ca_v_1.3 membrane targeting [31]. Ankyrin-B dysfunction also induces inappropriate afterdepolarizations, structural remodeling and arrhythmia in the atria with increased susceptibility to atrial fibrillation, ventricular fibrillation, [34,35] and has even been associated with WPW syndrome [36]. More recently, a loss-of-function mutation in ankyrin-B was identified in a patient with severe arrhythmogenic cardiomyopathy and sudden death [37]. The diversity of these phenotypes is likely the result of ankyrin-B mutations disrupting expression in subregions of the heart or subsets of interacting partners, while more complete disruption of ankyrin-B through heterologous knockdown in mice recapitulates a more comprehensive set of phenotypes [17,35,38,39,40].

Beyond transgenic models investigating variants or knockdown, ankyrin-B has also been identified to experience protein loss in human ischemic and non-ischemic heart failure, leading to concomitant loss in binding partners like NKA [25]. The protease inhibitor calpain has been found to preserve ankyrin-B and NKA at the border zone in a mouse model of ischemia-reperfusion and reduce infarct size. Thus, defects in ankyrin-B function give rise to a diverse array of abnormal excitability throughout multiple locales of the cardiac conduction system with important implications for human disease. 

### 3.2. Ankyrin-G

**Figure 4:**
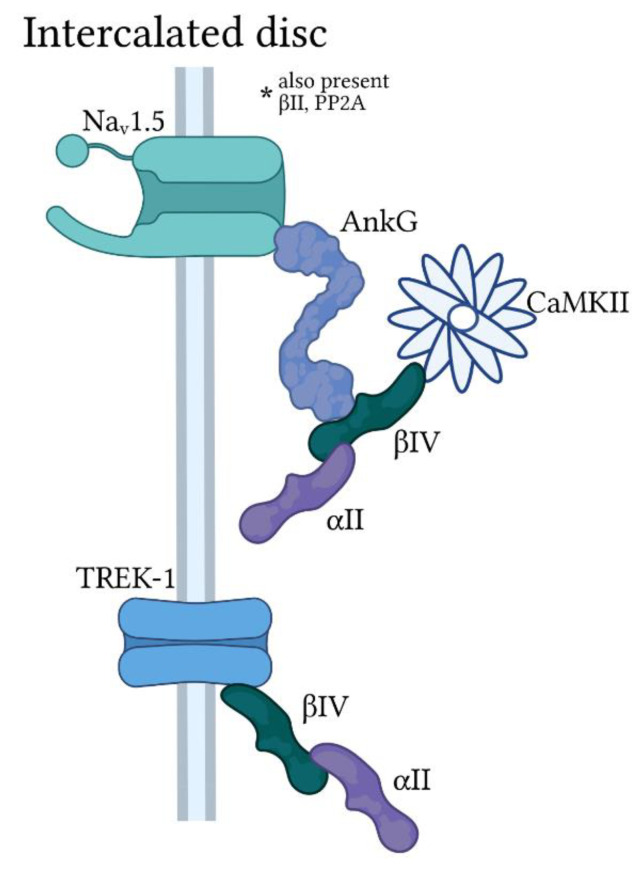
Ankyrin-G/spectrin-based macromolecular complexes at the cardiomyocyte intercalated disc. Ankyrin-G (AnkG) is found primarily at the cardiomyocyte intercalated disc with α_II_ and β_IV_-spectrin in a macromolecular complex for regulation of the voltage-gated Na^+^ channel Na_v_1.5 by Ca^2+^/calmodulin-dependent kinase II (CaMKII). A subpopulation of the protein phosphatase PP2A is also targeted by AnkG/spectrin for modulation of Na_v_1.5 activity (not pictured). β_IV_-spectrin also associates with the two-pore domain K^+^ channel TREK-1 at the intercalated disc in an AnkG-independent manner.

Ankyrin-G organizes macromolecular complexes to support conduction of the electrical impulse in neuronal and cardiac tissue, primarily through its association with voltage-gated Na^+^ channels (Na_v_) [41,42,43,44]. Specifically, ankyrin-G binds directly to a conserved motif found in DII-DIII linker of several Na_v_ α-subunits, including the predominant cardiac isoform Na_v_1.5. Ankyrin-G and Na_v_1.5 colocalize primarily at the intercalated disc membrane important for electrical and mechanical communication between neighboring myocytes with secondary expression at t-tubules or lateral membrane (Figure 2 and Figure 4). A host of loss-of-function mutations have been identified in *SCN5A* (encodes Na_v_1.5) and linked to a wide range of cardiac conduction disorders, including sick sinus syndrome, cardiac conduction disease, atrial fibrillation, and Brugada syndrome [11,45,46,47,48]. Beyond direct defects in Na_v_1.5, mutations in a large number of accessory proteins, including β-subunits, adapter, cytoskeletal and regulatory proteins, have been linked to cardiac conduction disorders and arrhythmia [49,50,51]. Relevant to ankyrins, a human mutation in the ankyrin-binding motif in Na_v_1.5 (E1053K) disrupts ankyrin-G/Na_v_1.5 interaction giving rise to loss of Na_v_1.5 membrane targeting and abnormal channel function. Specifically, HA-tagged E1053K Na_v_1.5 expressed in ventricular myocytes show minimal membrane surface expression compared to HA-tagged WT Na_v_1.5, which express readily at intercalated disc and t-tubule domains. Studies in heterologous cells support that the trafficking defect is likely not due to alterations in Na_v_1.5 folding or stability. Interestingly, the E1053K mutation is associated with Brugada syndrome, characterized by potentially fatal ventricular arrhythmias with specific ECG abnormalities in the absence of structural heart disease [43]. Cardiac-specific deletion of ankyrin-G in mice gives rise to loss of Na_v_1.5 membrane expression and activity with pronounced bradycardia together with evidence of atrial and ventricular conduction slowing at baseline and increased susceptibility to AV block and ventricular arrhythmia following flecainide challenge [18].

Beyond direct interaction with Na_v_1.5, ankyrin-G has been proposed to regulate cardiac conduction through association with mechanical adhesion proteins at the intercalated disc. While electrical coupling is supported by gap junctions comprised of connexin family members (Cx40, Cx45, and/or Cx43 in different layers of the conduction system), mechanical coupling and structural stabilization is provided by adherens junctions and desmosomes. Plakophilin, desmoplakin, and desmin are examples of desmosomal proteins that depend on ankyrin-G for proper expression and localization at the intercalated disc [52], which may explain development of dilated cardiomyopathy in cardiac-specific ankyrin-G knockout mice [18]. 

## 4. Spectrins in the Cardiac Conduction System

Spectrin is a critical component of the cytoskeleton in a broad distribution of metazoan cell types, including erythrocytes, neurons, beta cells, epithelial cells and cardiac myocytes [7,8]. Spectrin assembles as a heterotetramer of α- and β-subunits to form an extended chain to provide support for fragile membranes and effectively link membrane proteins/lipids to the actin-based cytoskeleton. Mammals express 2 α- and 5 β-subunits with α_I_, α_II_, β_I_, β_II_ and β_IV_ detected to varying degrees in cardiac myocytes. Canonical α-spectrin is comprised of 20 triple-helical repeats (spectrin repeats) and a C-terminal calmodulin-related domain (Figure 2). β-spectrin consists of a conserved N-terminal actin-binding region, multiple spectrin repeats (17 for every isoform except for β_V_-spectrin that has 30), and a C-terminal domain with multiple signaling motifs. Interaction with ankyrins occurs through a motif in β-spectrin repeat 15 that is highly conserved across isoforms. As discussed for ankyrin, isoform-specific macromolecular complexes are achieved at least in part through distinct subcellular localization profiles of ankyrins and spectrins. There is growing appreciation for the multifunctional nature of spectrin family members beyond providing structural support for the cell membrane. For example, spectrins support long-range cellular communication involving, in part, coordination of signaling nanodomains for ion channels [42,53,54,55,56,57,58,59,60]. More recently, it has been discovered that spectrins modulate gene expression to control remodeling of cell function in response to chronic stress stimuli, although the precise mechanisms remain to be determined [61,62,63]. Thus, spectrins have evolved to serve a host of unique challenges faced by metazoan cells.

Cardiomyocytes express several different spectrin isoforms with distinct subcellular localization profiles, similar to the situation for the different ankyrin isoforms. α_II_-spectrin is widely expressed at membrane domains throughout the myocyte, while α_I_-spectrin is mostly restricted to lateral membranes (Figure 3 and Figure 4). In contrast, β-spectrins appear to be more domain specific with β_I_-spectrin found mostly at the lateral membrane, β_II_-spectrin at the z-line in close proximity to sarcoplasmic reticulum (SR) and t-tubule membranes (secondary expression at intercalated disc), and β_IV_–spectrin almost exclusively expressed at intercalated disc membrane (Figure 4).

Several reports have identified loss of function mutations in *SPTAN1* [64] and *SPTBN4* [65,66,67] in patients displaying a wide spectrum of neurodevelopmental phenotypes, central deafness, and motor dysfunction. While cardiac phenotypes have not been reported in these patients, studies in mice with similar loss of function result in both electrical and structural phenotypes associated with altered ion channel localization and expression (described below). It is possible that the severity of other neurological phenotypes overshadow cardiac complications in these patients. At the same time, a human mutation in *ANK2* has been shown to alter interaction with β_II_-spectrin resulting in severe cardiac arrhythmia [68], supporting a role for spectrin proteins in human cardiac pathophysiology.

### 4.1. α_II_-Spectrin

α_II_-spectrin (encoded by *SPTAN1*) is expressed in tissues throughout the body, including heart. Within the cardiomyocyte, α_II_-spectrin is found at lateral membranes, z-lines and the intercalated disc membrane [69]. Global α_II_-spectrin knockout in mice is embryonic lethal with pronounced malformation of multiple tissues including heart [70]. Cardiac-specific α_II_-spectrin knockout mice, on the other hand, are viable but show defects in cardiac electrical and mechanical function with evidence of conduction disturbances in the atria and ventricles [71]. α_II_-spectrin deficient myocytes show a significant decrease in *I*_Na_ together with similar decrease in transient outward K^+^ current *I*_to_. Interestingly, the cytoskeleton regulatory proteins Mena and VASP have been reported to interact with a specific α_II_-spectrin splice variant (SH3i) at z-line and intercalated disc [72]. Double deficiency of Mena and VASP leads to loss of structural integrity of these critical membrane domains and evidence of intra-atrial and intra-ventricular conduction slowing [72].

### 4.2. β_II_-Spectrin

Similar to α_II_-spectrin, β_II_-spectrin (encoded by *SPTBN1*) is widely distributed in tissues throughout the body and is the predominant β-spectrin isoform found in cardiomyocytes. β_II_-spectrin associates with a host of critical ion channels, transporters and regulatory molecules through its interaction with ankyrin-B (discussed previously). Global deletion of β®_II_-spectrin in mice is embryonic lethal reflecting its wide distribution and important role in development [73]. Cardiac-specific β_II_-spectrin knockout mice are viable with pronounced ventricular arrhythmia and even sudden death following acute catecholaminergic challenge [68]. Interestingly and relevant to the discussion of the cardiac conduction system, in addition to ventricular arrhythmias and remodeling, β_II_-deficient mice show bradycardia at baseline with increased heart rate variability and episodes of AV block. At the myocyte level, β_II_-spectrin deficiency induces abnormal intracellular Ca^2+^ homeostasis with increased frequency of inappropriate, spontaneous SR Ca^2+^ release events and action potential afterdepolarizations that serve as potential arrhythmia triggers. Altered myocyte Ca^2+^ handling and excitability is likely related to loss in expression/membrane targeting of ankyrin-B and associated transporters NCX and NKA. Interestingly, β_II_-deficient myocytes also showed disorganized RyR2 subcellular localization and decreased RyR2 expression, although the mechanism remains unknown.

### 4.3. β_IV_-Spectrin

β_IV_-spectrin (encoded by *SPTBN4*) is highly expressed in brain and plays a critical role with ankyrin-G in Na_v_ clustering at axon initial segments and nodes of Ranvier [74,75,76]. Thus loss of β_IV_-spectrin function is associated with defects in neuronal excitability in mice with prominent ataxia. In cardiomyocytes, β_IV_-spectrin is expressed almost exclusively at the intercalated disc membrane [18,55]. As in neurons, β_IV_-spectrin associates with ankyrin-G and Na_v_ (primarily Na_v_1.5) to orchestrate a platform for regulation of myocyte membrane excitability. While β_IV_-spectrin is required for proper membrane targeting of Na_v_ in neurons, loss of β_IV_-spectrin in myocytes does not affect membrane expression but rather disrupts channel regulation. Specifically, β_IV_-spectrin binds directly to Ca^2+^/calmodulin-dependent kinase II (CaMKII) and targets a kinase subpopulation with ankyrin-G/Na_v_1.5 at the intercalated disc. CaMKII phosphorylates Na_v_1.5 to modulate channel gating and the level of pathogenic so-called late current (*I*_Na,L_). Loss of β®_IV_-spectrin/CaMKII interaction prevents CaMKII-dependent phosphorylation of Na_v_1.5 at a specific residue in the DI-DII linker (Ser571) and abrogates stress-induced potentiation of *I*_Na,L_ [55,77,78]. Mutant mice expressing truncated β_IV_-spectrin lacking the CaMKII binding domain (*qv^3J^* mice) show normal excitability at baseline with resistance to sinus pause and premature ventricular contractions induced by acute adrenergic challenge. 

More recently, it was discovered that β_IV_-spectrin also regulates the cardiac conduction system through association with the two-pore domain K^+^ channel TREK-1 in cardiac myocytes. TREK-1 function has been well characterized in the nervous system with identified roles in nociception and depression phenotypes [79]. The channel is also expressed in heart across species, including mice and human [57,80,81,82,83]. Interestingly, β_IV_-spectrin is required for normal TREK-1 membrane expression in SAN and ventricular myocytes. Furthermore, loss of β_IV_-spectrin/TREK-1 interaction does not impact basal heart rate or conduction but promotes a dramatic increase in sinus pause in response to adrenergic stimulation. Consistent with this phenotype, cardiac-specific knockout of TREK-1 results in R-R prolongation, QTc prolongation, and increased incidence of sinus pause without major changes in baseline echocardiography features [75]. Notably, isolated SAN cells from these mice exhibit rapid spontaneous action potential firing, faster diastolic depolarization rate, depolarized maximum diastolic potential, and longer AP duration at 50% repolarization compared to SAN cells isolated from wildtype (WT) mice. TREK-1 has also been linked to defects in AV node conduction through its association with members of the Popeye domain containing (POPDC) family of cAMP effector proteins [84], implying a broader role for the channel in regulating function of the cardiac conduction system.

## 5. Conclusions

The cardiac conduction system is a heterogeneous collection of excitable tissues that govern generation and coordinated spread of electrical activation through the heart. Differential expression of ion channels is an important determinant of the precise sequence of events beginning with impulse generation in the SAN and ending with rapid spread of the activation wavefront through the ventricles. Defects in ion channel expression/function in myocytes throughout the conduction system have been linked to arrhythmia. More recently, there is growing interest in the important role for cytoskeletal and adapter proteins in organizing macromolecular complexes for regulation of ion channels and membrane excitability. While important gaps remain in our understanding of the dynamic role of the myocyte cytoskeleton, ankyrin and spectrin family members provide a clue about how these proteins function to regulate cardiac conduction and pathophysiology.

Ankyrins and spectrins work together to arrange macromolecular complexes important for regulation of action potential generation and propagation. Although a precise characterization of expression patterns is lacking, ankyrin and spectrin isoforms have been identified in myocytes throughout the conduction system with distinct roles through isoform specific interactions with a host of ion channels, transporters and regulatory molecules. Given that ankyrins show high degree of homology across key functional domains (same for spectrins), isoform-specific interactions are likely determined by distinct subcellular distribution for each family member. For example, ankyrin-B is found at distinct subcellular domains (lateral membranes, t-tubules) where it organizes macromolecular complexes important for normal Ca^2+^ homeostasis. Thus, defects in ankyrin-B-based pathways primarily impact cardiac pacemaking, and atrial and ventricular arrhythmia (mostly through increased likelihood of arrhythmia triggers, as opposed to conduction defects). On the other hand, ankyrin-G is found primarily at the myocyte intercalated disc with Na_v_1.5 to regulate membrane excitability. Arrhythmias related to defects in ankyrin-G/Na_v_1.5 are more closely associated with depressed excitability, slow conduction, or even block (exit block in case of SSS). Spectrins bind directly to ankyrin to link macromolecular complexes to the actin cytoskeleton and/or expand the functionality of these complexes by recruiting additional regulatory proteins. An important emerging role for spectrin family members is in modulation of gene expression through association with signaling pathways and transcription factors. For example, β_IV_-spectrin is found primarily at the cardiac intercalated disc with ankyrin-G/Na_v_1.5 but is not required for normal membrane targeting of the complex [18,55]. Instead, β_IV_-spectrin is responsible for recruiting CaMKII for regulation of Na_v_1.5 in response to adrenergic stress. Furthermore, β_IV_-spectrin binds directly to the transcription factor STAT3 providing a direct link between integrity of the cytoskeleton and gene expression [61,62]. A similar role has been identified for β_II_-spectrin in crosstalk with TGF- β signaling [68,73]. It is interesting to consider the possibility that spectrins have evolved to link integrity of macromolecular complexes at the membrane to gene programs important for adaptation to stress in myocytes throughout the conduction system.

Although the field has learned a great deal about control of cardiac impulse generation and propagation from studying ankyrin and spectrin family members, important questions remain. What lessons from ankyrins/spectrins may be extended to other cytoskeletal proteins (e.g., dystrophin or nuclear cytoskeletal proteins EMD and lamin [5])? How do acquired/inherited defects in cytoskeletal proteins manifest in cardiac pacemaking and/or conduction defects? What is the relationship between acute/chronic stress, status of the cytoskeleton and association proteins, myocyte gene expression and, ultimately, conduction? Can this knowledge be leveraged to precisely tune specific components of the conduction system? Going forward, it is anticipated that answers to these questions will lead to novel therapies to help the many human patients suffering from cardiac conduction disorders around the world.
